# Gene Therapy Targeting Nuclear Factor-κB: Towards Clinical Application in Inflammatory Diseases and Cancer

**DOI:** 10.2174/156652309788488569

**Published:** 2009-06

**Authors:** Sander W. Tas, Margriet J.B.M. Vervoordeldonk, Paul P. Tak

**Affiliations:** 1 Division of Clinical Immunology and Rheumatology, Academic Medical Center/University of Amsterdam, Meibergdreef 9, F4-105, 1105 AZ Amsterdam, The Netherlands; 2 Arthrogen BV, Amsterdam, the Netherlands

**Keywords:** Gene therapy, NF-κB, signal transduction, siRNA, decoy oligodeoxynucleotides, adenovirus, adeno-associated virus, adoptive transfer.

## Abstract

Nuclear factor (NF)-κB is regarded as one of the most important transcription factors and plays an essential role in the transcriptional activation of pro-inflammatory cytokines, cell proliferation and survival. NF-κB can be activated via two distinct NF-κB signal transduction pathways, the so-called canonical and non-canonical pathways, and has been demonstrated to play a key role in a wide range of inflammatory diseases and various types of cancer. Much effort has been put in strategies to inhibit NF-κB activation, for example by the development of pharmacological compounds that selectively inhibit NF-κB activity and therefore would be beneficial for immunotherapy of transplantation, autoimmune and allergic diseases, as well as an adjuvant approach in patients treated with chemotherapy for cancer. Gene therapy targeting NF-κB is a promising new strategy with the potential of long-term effects and has been explored in a wide variety of diseases, ranging from cancer to transplantation medicine and autoimmune diseases. In this review we discuss recent progress made in the development of NF-κB targeted gene therapy and the evolution towards clinical application.

## NUCLEAR FACTOR-κB SIGNALING PATHWAYS

The transcription factor nuclear factor-κB (NF-κB) is a key regulator of inflammation and therefore plays a key role in a wide range of inflammatory diseases [1].The mammalian NF-κB/Rel family has many members: RelA (p65), NF-κB1 (p50; p105), NF-κB2 (p52; p100), c-Rel and RelB. Each member, except for RelB, can form homodimers, as well as heterodimers with each other. The dimeric structure of NF-κB allows many different combinations to form, each exerting a distinct biologic function (reviewed in [2]). The main activated form of NF-κB is a heterodimer, consisting of a p50 or p52 subunit and the transactivating subunit p65. Inactive NF-κB resides in the cytoplasm associated with eight regulatory proteins called inhibitors of κB (IκB), of which IκBα, IκBβ and IκBε may be the most common. Importantly, the precursor proteins p100 and p105 can also function as IκB-like proteins. Different IκB proteins have distinct and overlapping specificities for NF-κB proteins and tissue distribution of IκBs may also differ, making them attractive targets for specific therapies [1]. For most known stimuli, degradation of IκBα is essential for release and activation of NF-κB. NF-κB can be activated via two different NF-κB signal transduction pathways.

**Fig. (1). Schematic representation of the NF-κB signal transduction pathways.**
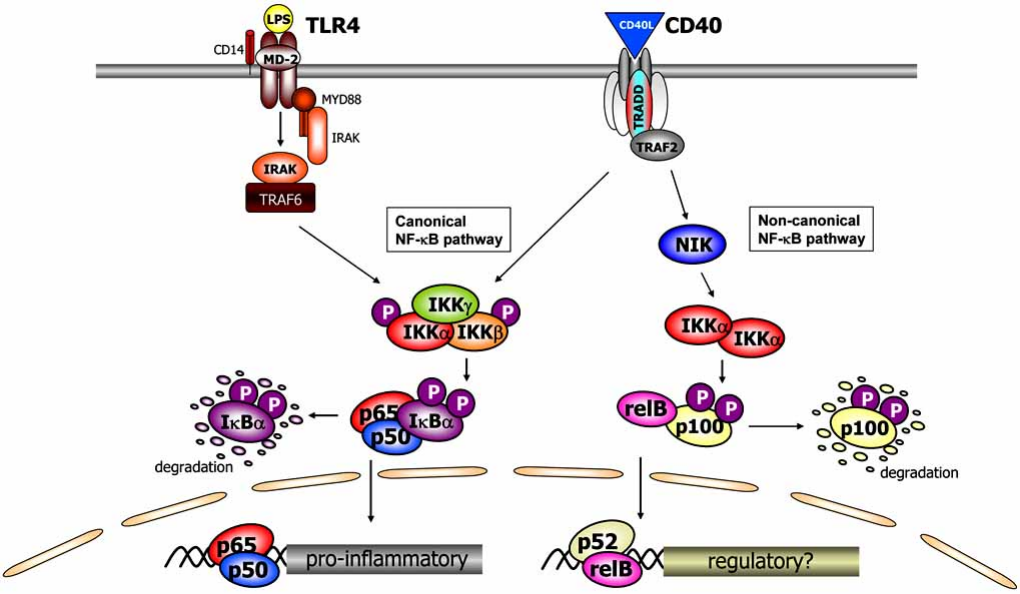
Nuclear factor-κB (NF-κB) can be activated by a multitude of different stimuli, like TNFα, LPS and CD40L. Activation of the canonical (also known as classical) pathway via Toll-like receptor (TLR) or cytokine receptor signaling depends on the IKK complex, which is composed of the kinases IKKα and IKKβ, and the regulatory subunit IKKγ (NEMO). Activated IKK phosphorylates (P) IκBα to induce its degradation by the 26S proteasome, allowing NF-κB dimers (p50-p65) to translocate to the nucleus and bind to DNA to induce NF-κB target gene transcription. Activation of the non-canonical (also known as alternative) pathway is strictly dependent on IKKα homodimers. The target for IKKα homodimers is NF-κB2/p100, which upon activation of IKKα by NIK is phosphorylated and incompletely degraded into p52, resulting in the release and nuclear translocation of p52-RelB dimers. This pathway can be triggered by the activation of members of the TNF-receptor superfamily such as CD40 (that also induce canonical NF-κB signaling), but not via pattern recognition receptors such as TLRs.

The canonical (also known as classical) NF-κB pathway requires activation of the inhibitor of κB (IκB) kinase (IKK) complex, consisting of the catalytic subunits IKKα (IKK1) and IKKβ (IKK2) [3,4], and the regulatory subunit IKKγ (or NEMO, NF-κB essential modifier) [5,6], followed by IKK-mediated degradation of the inhibitory IκB proteins. This results predominantly in the activation and nuclear translocation of the classical NF-κB dimer p50-RelA (Fig. **1**). Multiple signaling pathways that lead to NF-κB activation, like tumor necrosis factor (TNF)α signaling, Toll-like receptor (TLR) signaling and T cell receptor signaling, converge at the level of the IKK complex. In this pathway IKKβ is essential for NF-κB activation in response to pro-inflammatory stimuli [7-10], whereas IKKα is dispensable for IKK activation and induction of NF-κB DNA-binding activity in most cell types [10-12].

In contrast, the non-canonical (also known as alternative) pathway is strictly dependent on IKKα homodimers and does not require IKKβ and NEMO/IKKγ [13,14]. The target for IKKα homodimers is NF-κB2/p100, which is incompletely degraded into p52 upon activation of IKKα by NF-κB-inducing kinase (NIK), resulting in the release and nuclear translocation of p52-RelB dimers (Fig. **1**). This pathway can be triggered by the activation of members of the TNF-receptor superfamily such as the lymphotoxin β receptor, B-cell activating factor belonging to the TNF family (BAFF)-receptor and CD40L (that also induce canonical NF-κB signaling), but not via pattern recognition receptors such as Toll-like receptor 4 (TLR4), the receptor for LPS [15]. It has been suggested that the canonical and non-canonical NF-κB pathways play distinct roles in immunity (reviewed in [16]). It has been demonstrated that IKKα has an important function in thymic organogenesis for the establishment of central tolerance in cooperation with NIK [17]. However, the precise mechanisms involved have not been fully elucidated yet.

The non-canonical pathway also appears to have an immunoregulatory role in addition to its role in developmental biology [12,18-20]. IKKα negatively regulates inflammation in macrophages via either control of IKKβ activity [21] or by accelerating the turnover of pro-inflammatory RelA and c-Rel-containing dimers and their removal from pro-inflammatory gene promoters [22]. In addition, NIK has a role in the development of regulatory T cells (Treg)[23]. Furthermore, we found that selective knock-down of the non-canonical pathway using siRNA for IKKα or NIK in dendritic cells (DC) resulted in increased pro-inflammatory cytokine production [24], suggesting that a similar negative regulation also takes place in DC. Recent literature demonstrates that the non-canonical NF-κB pathway is also required for other regulatory functions in these cells, including the induction of Treg and the immunoregulatory enzyme indoleamine-2,3-dioxygenase (IDO) [24,25]. Based on these findings it is hypothesized that non-canonical NF-κB signaling is important in the regulation of immune responses [26].

Another mechanism by which transcription of NF-κB responsive genes can be regulated is via modification of histone acetylation by histone acetyltransferases (HATs) and histone deacetylases (HDACs) [27]. Histone acetylation status influences the accessibility of DNA to the transcriptional machinery by changing the folding and functional state of the chromatin fiber [28]. NF-κB interacts with HATs to positively regulate gene expression and with HDACs to negatively regulate transcription of NF-κB responsive genes [29]. Recently, a novel mechanism of p65 transcriptional regulation was described as pro-inflammatory stimuli activate IKKα-mediated sumoylation-dependent phosphorylation of PIAS1. This results in the repression of NF-κB- and STAT1-dependent transcriptional responses [30]. These and other regulatory mechanisms are described in great detail in an excellent recent review article [31].

## NF-κB INHIBITION: GENE THERAPY VS. PHARMACOLOGICAL INHIBITORS

NF-κB plays a key role in the expression of pro-inflammatory genes and is abundant in a wide variety of inflamed tissues like rheumatoid arthritis (RA) synovium and colonic epithelium in inflammatory bowel diseases [1,32,33]. NF-κB not only induces the transcription of pro-inflammatory cytokines [34-36] and chemokines [37], but also regulates the expression of cell adhesion molecules like E-selectin, vascular cell adhesion molecule (VCAM)-1 and intercellular adhesion molecule (ICAM)-1 [38,39], indicating an important role in leukocyte adhesion and transmigration resulting in accumulation of immune cells at sites of inflammation. NF-κB also functionally interacts with other pathways and transcription factors, like activator protein 1 (AP-1) to coordinate stimulation of matrix metalloproteinase (MMP) production leading to tissue destruction [40,41]. In many cell types NF-κB also plays an anti-apoptotic role [42-44], which may be regulated via Akt, the suppression of caspase-8 activation or IKKβ related functions [7,45,46].

Because of its pivotal role in inflammation and cell proliferation a lot of attention has been given to strategies that inhibit NF-κB activity. NF-κB inhibition may be beneficial in wide variety of diseases including cancer, many immune-mediated inflammatory diseases and the prevention of transplant rejection. NF-κB activity can be targeted at virtually every step of the signaling cascade(s) that lead to NF-κB activation. However, the biologic consequences may vary widely, depending on the level of disruption in the signal transduction pathway, since more and more data indicate that complex cross-talk with other signaling pathways exists. Therefore, it is conceivable that with an increasing understanding of the function of individual NF-κB subunits, IκB proteins, and kinases in different cell types and their contribution to the pathogenesis of different diseases, one might attain therapeutic efficacy with minimized systemic toxicity by selectively targeting proteins that play a pivotal role in a diseased tissue, allowing normal function of other proteins.

Interestingly, many of the drugs that are currently used for treating inflammatory conditions like non-steroidal anti-inflammatory drugs (NSAIDs), disease-modifying anti-rheumatic drugs, cyclosporine A and corticosteroids have inhibitory effects on NF-κB activity (reviewed in [47]). It should be noted, however, that these drugs lack specificity for inhibiting NF-κB activity and consequently require relatively high concentrations, raising the issue of toxicity and adverse events. Therefore, much effort has been put in the development of highly specific pharmacological NF-κB inhibitors [48-50]. Most of these targeted, more specific NF-κB inhibitors exert their action at the level of the IKK complex (mainly IKKβ) or IκBα. Recent literature suggests that IKKβ inhibition is particularly beneficial in chronic inflammatory conditions [51,52]. Small molecule IKKβ inhibitors are more selective and might cause less severe side effects than other systemic approaches targeting NF-κB activity.

Various systemic and local pharmacological approaches to specifically inhibit the activation of this transcription factor *in vivo* by targeting the IKK complex have proven very successful in the amelioration of inflammation in animal models of diseases like arthritis or multiple sclerosis [53-57], and other diseases such as cancer [48,50,58-61]. Although some of these compounds display highly specific NF-κB blocking activity, these pharmacological inhibitors will not be discussed here, as this review is primarily focused on gene therapy, but they are extensively discussed in recent review articles [56,62]. So far, no potent specific IKKα inhibitors have been described.

In comparison with pharmacological inhibitors the application of gene therapy to target NF-κB has several advantages, especially in chronic immune-mediated inflammatory diseases [63]. Gene therapy can offer a sustained (in theory life-long) beneficial effect, resulting in long term action without the need of frequent re-administration of a recombinant protein. Therapeutic vectors can be administrated either systemically or locally at the site of inflammation, the latter approach reducing the risk of toxic side-effects and resulting in constant therapeutic levels in the desired target tissue. Gene therapy targeting proteins involved in signal transduction has some potential limitations. Since signal transduction molecules are expressed intracellularly, this type of construct should preferably be expressed in all target cells in order to exert maximal effect, whereas introduction of a gene encoding a secretory therapeutic protein only requires transduction of a stable cell population at the target site to ensure continuous production and consequently exerts its effects also on non-transduced cells [63,64]. Furthermore, compared to low-molecular compounds that target NF-κB in virtually all cell types, a gene therapeutic approach may not reach all preferred cell types since viral vectors require certain specific receptors for cell entry. On the other hand, it can also be advantageous to target specific cells in order to reduce unwanted side-effects. In addition, gene therapy mostly uses viral vectors that may evoke immune responses resulting in limited transgene expression. However, these limitations may be circumvented by choosing the right vector and optimal promoter for a specific target tissue (see below).

## GENE THERAPEUTIC STRATEGIES TARGETING NF-κB

### Strategies Using Viral Vectors

Viral-mediated gene transfer is currently the most efficient system for delivering therapeutic proteins *in vivo* [63-65]. There is a continuous need for optimizing vectors for gene therapy in order to achieve highly efficient transduction of the target tissue and to reduce immune responses, to ensure stable expression of the therapeutic transgene over time. These topics are mostly defined by the route of administration and tropism of the vector, i.e. the cell type(s) that a certain viral vector is capable to transduce. Therefore, the type of vector should be chosen carefully based on the cell types/tissue that will be targeted and the nature of the disease, in order to achieve maximal therapeutic effects. Tissue-specific and disease-regulated transgene expression (for example by using an NF-κB responsive promoter) could also further improve the overall safety of gene therapy approaches. The use of these promoters that are only active in the target cell or are regulated by pharmacological systems or physiological stimuli has been described in several reviews [63,66-69] and research is still ongoing to further improve such promoters. In future pre-clinical and clinical studies it must be determined if the use of such promoters is applicable and advantageous in human subjects.

#### Adenoviral Vectors

Adenoviruses have certain features, which make them attractive vectors for gene transfer to target cells. Some of these characteristics include their ability to infect a broad range of cell types, including dividing as well as non-dividing cells, the ease with which the adenovirus genome can be manipulated, and the ability to obtain high titers. However, although gene therapy with adenoviral vectors has proven to be efficient for target validation in animal models of disease, adenoviral vectors are not widely used in clinical trials for chronic diseases because they may evoke serious host immune responses and as a consequence give only transient expression of the transgene [63,70]. For the treatment of tumors, however, adenoviral vectors are widely utilized for clinical application, since life-long expression is not required because the strategy is aimed at reducing or even eliminating the tumor.

In the past one death has been contributed to gene therapy using an adenoviral vector: in 1999 a patient suffering from an X-linked genetic disease of the liver (ornithine transcarbamylase deficiency) died after receiving an adenovirus containing the corrective gene. The virus triggered a massive immune response, leading to multiple organ failure and brain death [71]. The Food and Drug Administration (FDA) investigation of this case of death concluded that the scientists involved in the trial broke several rules of conduct. They did not report that two patients who had already been treated in this trial had experienced serious side effects from the gene therapy and it was not mentioned in the informed consent documentation that monkeys given a similar treatment died in the study. If these data had been available the death, in this case related to the study drug, could most likely have been prevented [72].

Adenoviral vectors targeting NF-κB have been used *in vitro* and in a variety of animal models. A pioneering study employing intra-articular injection with a dominant-negative adenoviral IKKβ construct (Ad.IKKβdn) in rats with adjuvant arthritis showed a reduction of NF-κB nuclear translocation in cells of the synovial tissue and a significant decrease in paw swelling [52]. Inhibition of IKKβ also resulted in reduced pro-inflammatory cytokine production in synovial tissue [73]. Furthermore, Ad.IKKβdn has demonstrated to potently inhibit the response of human endothelial cells to inflammatory stimuli [74]. In addition, this construct exhibited anti-inflammatory effects in primary human airway smooth muscle cells [75] and reduced IL-13-induced tissue inflammation, fibrosis and alveolar remodelling in a mouse model of asthma [76]. Ad.IKKβdn was also tested as anti-cancer therapy and sensitized human prostate carcinoma cells, neuroblastoma cells, and lung cancer cells to TRAIL- or TNF-induced apoptosis [77-79]. From these experimental data it can be concluded that targeting NF-κB at the level of IKKβ appears promising in inflammatory conditions as well as cancer.

Adenoviral vectors carrying an IκBα super-repressor (Ad.IκBαSR) have been used extensively, predominantly *in vitro* in cell types associated with inflammatory conditions [75,80-88]. Blocking the NF-κB pathway by Ad.IκBαSR resulted in suppressed constitutive and TNFα-induced NF-κB activity and increased sensitivity to pro-apoptotic stimuli *in vitro*, both in normal human macrophages and in RA synovial cell cultures and macrophages [34,43,89]. Ad. IκBαSR also inhibited the spontaneous production of TNFα and other pro-inflammatory cytokines in cultured explants of rheumatoid synovial tissue and inhibited the production of MMPs 1 and 3 while not affecting their tissue inhibitor [90]. Studies in osteoarthritis synovial cells also resulted in reduced production of inflammatory and destructive mediators [91,92].

An interesting *in vivo* model in which adenovirus-mediated IκBα over-expression was shown to have beneficial effects is ischemia-reperfusion injury in rats. This was demonstrated in experimental lung transplantation [93], after myocardial infarction [94], and in liver ischaemia/reperfusion injury [95, 96]. In addition, this approach was also successful in the prevention of post-angioplasty lumen loss in a rabbit iliac artery restenosis model [97].

Accordingly, Ad.IκBαSR decreased hepatocyte proliferation in a rat model of obstructive jaundice [98]. Interestingly, adenoviral IκBα over-expression may also improve wound healing in rats, demonstrated by increased collagen deposition due to decreased inflammation [99]. In addition to targeting inflammation, adenovirus-mediated IκBα gene transfer has been applied to improve the sensitivity of various tumors to anticancer drugs or radiation both *in vitro* and *in vivo* in pre-clinical models by increasing apoptosis [100-109].

#### Adeno-Associated Virus

Adeno-associated virus (AAV) has emerged as a potential novel vector that lacks many of the immunogenic characteristics of adenoviral vectors and appears to be safe [110]. AAV is a single-stranded DNA virus that, compared to adenoviral vectors, induces a significantly reduced immune response and is not associated with disease in humans. Recombinant (r)AAV vectors typically remain episomal as concatemers and integrate only at very low frequency throughout the genome. Therefore, the risk of activation of oncogenes is considered extremely low, although a recent study identified AAV vector integration sites in mouse hepatocellular carcinoma that developed after β-glucuronidase gene therapy, suggesting insertional mutagenesis is possible [111]. rAAV vectors have gained much attention due to their ability to mediate efficient transduction of both dividing and non-dividing cells and their capability to induce long-term gene expression in the absence of toxicity in a variety of tissues. Recently, transgene expression was demonstrated in dog muscle tissue for over 8 years [112]. Moreover, AAV gene therapy has become more feasible as a consequence of the improvement of the production of clinical grade AAV vectors, resulting in production of large vector quantities. In addition, efforts to produce clinical-grade empty-capsid free vector batches have met with success [113] and should significantly reduce the antigen load, because many early trials and even current clinical vector batches contain a full : empty capsid ratio of 1 : 3 to 1 : 100 (only the full capsids contain the transgene). Although rAAV generally transduces cells less efficiently than an adenoviral vector expressing the same transgene, the stable longterm expression of the transgene makes it an attractive candidate for treating chronic inflammatory diseases [63,114]. In total at least 46 clinical trials have been conducted or are in progress with rAAV vectors carrying different transgenes, all showing a good safety profile. One subject enrolled in an RA trial receiving systemic anti-TNF therapy in combination with an rAAV2 vector expressing a TNF-blocking agent locally in the joint developed fatal disseminated histoplasmosis. However, after careful evaluation this tragic event was ultimately considered unrelated to the study agent [115].

Recently, we compared the efficiency of five different rAAV serotypes (rAAV1-rAAV5) to transduce arthritic synovium in both mouse and rat models of arthritis. We demonstrated that rAAV5 is an excellent potential vector for local gene therapy in patients with RA, allowing long-term expression of the transgene [116,117]. Importantly, although 25% to 60% of humans have neutralizing antibodies against different AAV serotypes, RA patients exhibit only low titers of neutralizing antibodies against AAV5 compared to AAV2 (Vervoordeldonk *et al.*, unpublished results), and these low titers are not anticipated to interfere with local transduction in the joint. As a next step towards development of gene therapy for arthritis, we constructed an rAAV5 vector containing the IKKβdn transgene (AAV5.IKKβdn) and demonstrated that local NF-κB blockade by IKKβdn using rAAV5 as a vector significantly reduced established arthritis *in vivo* in rats*, *resulting in a significant reduction of synovial inflammation. Importantly, we also unambiguously showed that rAAV5 can be used to target NF-κB in human synovial tissue *ex vivo*, resulting in reduced TNFα-induced IL-6 production when AAV5. IKKβdn was used to inhibit NF-κB [118]. Local rAAV2-mediated gene transfer of IκBα has been demonstrated to limit infarct size in a mouse model of myocardial ischemia-reperfusion injury [119] and to reduce neointimal hyperplasia induced by flow cessation in the mouse carotid artery, suggesting that rAAV-mediated gene transfer of IκBα might represent a novel therapeutic approach for the treatment of restenosis [120].

Taken together, these limited data on the feasibility of gene therapy targeting NF-κB using rAAV vectors are very promising, as these vectors are currently almost certainly the most attractive candidates for treating (chronic) inflammatory diseases, because of the stable longterm expression of the transgene. However, more research should be done on vector optimization to improve transduction of target cells.

#### Other Viral Vectors

Compared to adenoviral and AAV vectors, not many studies have been performed targeting NF-κB using other viral vectors such as lentivirus or retrovirus. Retroviral vectors have been used extensively in the laboratory and in the majority of gene therapy clinical trials. Most retroviral vectors are based on the Moloney murine leukemia virus (MMLV) and have as major limitation their inability to infect non-dividing cells. MMLV-based vectors are usually employed for *ex vivo* gene therapy. A possible drawback of this virus is the random manner in which retroviruses can integrate into the host genome and can induce insertional mutagenesis leading to pathology, potentially including malignancies.

Lentiviral vectors are based on complex retroviruses (lentiviruses) such as human immunodeficiency virus (HIV). There are several advantages with the use of a lentiviral vector. The vector has a relatively high cloning capacity, the production process of the vector is relatively simple and the host-inflammatory reactions are moderate. Moreover, in contrast to murine retroviral vectors, lentiviral vectors transduce a variety of quiescent cells very efficiently. Over the past years it has been shown that lentiviral vectors mediate efficient transduction of various cell types *in vitro* and *in vivo*. However, more development is required to employ these vectors for clinical applications.

In a pioneering *in vitro* study the feasibility of suppression of inflammatory responses in appropriate target cells (monocytic THP-1 and immortalized human endometrial stromal cell lines) by suppression of NF-κB activity was established using a retroviral vector overexpressing IκBα [121]. Another, more recent, interesting study demonstrated that lentiviral-mediated IκBα overexpression in dorsal spinal cord glia attenuates sciatic nerve injury-induced neuropathic pain in the rat [122].

### Non-Viral Strategies

#### NF-κB Decoy Oligodeoxynucleotides

One of the first methods described to regulate NF-κB gene expression was the use of synthetic double-stranded DNA oligodeoxynucleotides (ODN) containing the NF-κB target sequence that can be introduced *in vivo* as “decoy” cis elements to bind the transcription factor and thereby interfere with binding of NF-κB to promotor regions in genes [123]. This method has been used extensively to specifically inhibit NF-κB activity in numerous cells *in vitro*, as well as in many animal models of inflammation, transplant tolerance, ischaemia-reperfusion injury, and cancer (reviewed in [124,125]). Due to limitations in space, we will only focus on several highlights of this approach in this review.

Revolutionary work by Morishita and colleagues has demonstrated that *in vivo* transfection of NF-κB decoy ODN reduced the extent of myocardial infarction following reperfusion in rats [123]. Of note, this method also proved to attenuate in-stent restenosis in cardio-vascular medicine both in a rabbit model [126] and in humans [127,128].

NF-κB blockade with NF-κB decoy ODN has also been shown to inhibit the development of arthritis and joint destruction in various animal models of arthritis [44,129,130], and to inhibit NF-κB activity *ex vivo* in human synovial cells derived from patients with RA [131]. Interestingly, this method may also be successful in the amelioration of osteoporosis through inhibition of osteoclast activation and differentiation, as demonstrated by a study in rats [132]. In addition, this technique has been proven to reduce inflammation in mouse models of allergic airway disease [133], inflammatory bowel disease [134,135] and endotoxic shock [136], as well as in glomerulonephritis both in mice and rats [137, 138]. When applied locally NF-κB decoy ODN have been demonstrated to reduce skin inflammation [139,140]. Another interesting area in which decoy ODN were tested is transplant tolerance. NF-κB decoy ODN reduced inflammation and prolonged graft survival in a rat renal allograft model [141,142] and a rat model of lung transplantation [143]. Furthermore, decoy ODN were tested in several mouse models of cancer. NF-κB decoy ODN inhibited cachexia in a mouse tumor model when injected intratumorally [144], inhibited hepatic metastasis when infused intravenously [145], and sensitized colon cancer liver metastases to paclitaxel-induced apoptosis [146].

Taken together, NF-κB inhibition with NF-κB decoy ODN has generated promising results. However, this strategy certainly also has limitations for wide application in human diseases, because the therapeutic potential of decoy ODN is unclear as these molecules have a short half-life and need to be administered frequently, especially in chronic diseases. In addition, decoy ODN are quite large and polar, which will likely hinder their cellular uptake and bioavailability [62].

#### Small-Interfering (si)RNA

Since its discovery in 1998 [147] RNA interference (RNAi) has attracted a great deal of interest, including a Nobel prize [148,149], in particular after it was demonstrated that double stranded small interfering RNAs (siRNA) could trigger RNAi in mammalian cells [150]. This discovery revealed a role for RNA in the regulation of gene expression, in addition to its traditional role in transferring genetic information. Consequently, siRNA provided a valuable basic research strategy for studying the biological function of a gene by selective knock-down and, perhaps even more exciting, offered the possibility to develop a powerful new class of therapeutics. Development of therapeutics using siRNA resulted already in five different clinical trials that are ongoing and several more poised to enter the clinic in the coming years. In the RNAi pathway larger dsRNA molecules are processed by the enzyme Dicer into shorter siRNAs that are typically 21-23 base pairs in length and complementary to specific mRNA sequences. The siRNA duplexes bind to a larger, multi-protein RNA-induced silencing complex (RISC). Subsequently, the sense strand is degraded, resulting in hybridization of the anti-sense strand with the complementary sequence in the target mRNA. Ultimately, this leads to cleavage of the mRNA strand by an enzyme of the RISC, which prevents translation and results in posttranslational silencing of gene expression [151]. Although this technique promises to be a very powerful new treatment option for many diseases, the optimal *in vivo* delivery system has to be determined yet and this is the subject of intensive research (reviewed in [152]). Approaches that are tested include injection of “naked” siRNA, non-viral [153] or viral delivery methods [154,155](see below).

Although the phenomenon was only discovered recently, RNAi has been used extensively to target NF-κB *in vitro* resulting in a vast amount of interesting data. Diseases for which NF-κB inhibition via p65 or p50 siRNA may be considered include rheumatoid arthritis [156], osteoarthritis [157], esophageal cancer [158], head and neck squamous cell carcinoma [159,160], colorectal cancer [161], and myelodysplastic syndrome [162].

Significant progress has been made in recent years in the delivery of siRNA *in vivo *using non-viral methods, and several promising siRNA delivery platforms have begun to emerge. These platforms include liposomes, in which siRNA is encapsulated in a lipid vesicle; polyplexes, in which a cationic carrier is used to bind siRNA to form siRNA-containing nanoparticles; liposome-polycation-DNA (LPD) complexes, in which an siRNA-containing polyplex is encapsulated in a lipid vesicle; and siRNA conjugates, in which siRNA is coupled to a targeting moiety that carries the siRNA into target cells via receptor-mediated endocytosis [155]. For siRNA therapeutics to achieve their full potential as a revolutionary class of drug molecules, multiple distinct delivery technologies will probably be needed, with selection of the delivery approach being dependent on the nature of the clinical indication, the route of administration to be used, and the cell types to be targeted.

Only a few papers describe non-viral siRNA based strategies to inhibit NF-κB activation in animal models of diseases. Lipid based siRNA-mediated knock-down of p65 via intraperitoneal administration in combination with paclitaxel prolonged survival in a mouse model of peritoneal metastasis of gastric cancer [163], suggesting that this approach may also be beneficial in humans via sensitization of tumor cells to chemotherapy. Recently, it was demonstrated that siRNA-mediated reduction of IKKβ prevented TNFα-induced insulin resistance in human skeletal muscle *ex vivo* [164], possibly reducing insulin resistance.

### Combinatorial Approaches

The aforementioned techniques to regulate NF-κB activity are of course not mutually exclusive and can be combined, for instance by constructing a viral vector expressing siRNA or via adoptive transfer of cells (i.e. dendritic cells) genetically modified *ex vivo* to ensure NF-κB inhibition only in the desired cell type.

#### Viral Vectors Expressing siRNA

The half-life of unmodified siRNA *in vivo* is short due to rapid elimination by the kidney and degradation by endogenous serum RNases (1-60 minutes). To overcome the limitation of *in vivo* RNAi, several viral vectors are used as an alternative method due to their infective properties to effectively deliver short hairpin siRNAs (shRNAs) resulting in long term silencing. In rats adenovirus-mediated expression of p65-specific shRNA has been demonstrated to suppress early experimental osteoarthritis after intra-articular injection [165]. Furthermore, rAAV2 coding for shRNA targeting p65 reduced TNFα-induced IL-8 production in human bronchial epithelial cells *in vitro *[166], but *in vivo *studies have not been reported yet. Using an adenoviral expression system to deliver a somewhat different RNA-based construct (aptamer) that specifically binds p50, it was demonstrated that human non-small cell lung cancer cells are sensitized to chemotherapy both *in vitro* and *in vivo* in a lung tumor xenograft mouse model [167]. Interestingly, RNAi through lentiviral delivery of shRNA against p65 prevented cardiac hypertrophy and heart failure in a mouse model after direct delivery into the heart [168].

#### Adoptive Transfer of Genetically Modified Cells

Another method to obtain long term inhibition of NF-κB in the desired cell type is via adoptive cellular transfer, in which cells are genetically modified *ex vivo *resulting in reduced NF-κB activation in the target cell. At present, there is a lot of interest in cell-based therapies for inflammatory disorders and cancer, especially in the *ex vivo* manipulation of DC to induce tolerance leading to remission or immuno-regulation of autoimmune diseases [169-173]. Recently, it has been described that cellular immunotherapy using DC in which the canonical NF-κB pathway was selectively blocked *ex vivo* by adenoviral dominant-negative IKKβ gene therapy resulted in immunoregulation both *in vitro* [174,175] and in the formation of potent CD4^+^ Treg that prevented transplant rejection *in vivo* in a rat model of kidney allotransplantation [176,177]. In another study, adoptive transfer of *ex vivo* siRNA-mediated RelB-silenced DC resulted in prevention of allograft rejection in murine heart transplantation [178]. Similarly, blocking of DC maturation by the combination of NF-κB ODN with an adenoviral vector encoding CTLA4-Ig *ex vivo* resulted in prolonged cardiac allograft survival after adoptive transfer of these cells [179]. Altogether, these results indicate that this approach could have beneficial effects in immune-mediated inflammatory diseases as well.

The experimental approaches to generate DC for immunotherapy described above are based on *in vitro* generation of tolerogenic DC via gene therapy-mediated NF-κB inhibition, followed by intravenous administration of the DC. This technique has several possible disadvantages for application in the clinic: (I) generation of DC from monocytes requires a minimum of 5-7 days culture *in vitro*, which may lead to genetic alterations in the cells; (II) tolerogenic immature DC are unstable and easily mature *in vitro* and *in vivo,* which could cause adverse effects in autoimmune diseases; (III) only a very small fraction of the injected DC traffic to secondary lymphoid organs and interact with naïve T cells (reviewed in [180]). To avoid these potential drawbacks, it would be extremely valuable to develop techniques to generate tolerogenic DC via *in situ* targeting of DC *in vivo*. Unfortunately, until now no successful procedure has been discovered. Therefore, a lot of research needs to be done to create methods that allow specific targeting of siRNA or viral vectors to DC* in vivo*.

## CONCLUSION

The strategies aimed at interfering with NF-κB signal transduction that are reviewed in this paper provide the tools for more effective and more specific blockade of signaling molecules, resulting in precisely defined biological effects. The ultimate benefit of targeting NF-κB will obviously depend on the delicate balance between suppressing inflammation and interfering with normal cellular functions. By using local gene therapy or optimizing systemic targeted approaches to specifically interfere with signal transduction in target cells, this goal appears attainable. Future studies on disease-regulated and/or tissue-specific promoters in gene therapy using viral vectors, strategies to limit immune responses to vectors, and new techniques to efficiently deliver siRNA *in vivo* will guide the way towards clinical application of gene therapy targeting NF-κB.

## References

[1] Tak PP, Firestein GS (2001). NF-kappaB: a key role in inflammatory diseases. J Clin Invest.

[2] Hayden MS, Ghosh S (2008). Shared principles in NF-kappaB signaling. Cell.

[3] DiDonato JA, Hayakawa M, Rothwarf DM, Zandi E, Karin M (1997). A cytokine-responsive IkappaB kinase that activates the transcription factor NF-kappaB. Nature.

[4] Zandi E, Chen Y, Karin M (1998). Direct phosphorylation of IkappaB by IKKalpha and IKKbeta: discrimination between free and NF-kappaB-bound substrate. Science.

[5] Rothwarf DM, Zandi E, Natoli G, Karin M (1998). IKK-gamma is an essential regulatory subunit of the IkappaB kinase complex. Nature.

[6] Yamaoka S, Courtois G, Bessia C (1998). Complementation cloning of NEMO, a component of the IkappaB kinase complex essential for NF-kappaB activation. Cell.

[7] Li ZW, Chu W, Hu Y (1999). The IKKbeta subunit of IkappaB kinase (IKK) is essential for nuclear factor kappaB activation and prevention of apoptosis. J Exp Med.

[8] Senftleben U, Li ZW, Baud V, Karin M (2001). IKKbeta is essential for protecting T cells from TNFalpha-induced apoptosis. Immunity.

[9] Li Q, Van Antwerp D, Mercurio F, Lee KF, Verma IM (1999). Severe liver degeneration in mice lacking the IkappaB kinase 2 gene. Science.

[10] Delhase M, Hayakawa M, Chen Y, Karin M (1999). Positive and negative regulation of IkappaB kinase activity through IKKbeta subunit phosphorylation. Science.

[11] Chu WM, Ostertag D, Li ZW (1999). JNK2 and IKKbeta are required for activating the innate response to viral infection. Immunity.

[12] Hu Y, Baud V, Delhase M (1999). Abnormal morphogenesis but intact IKK activation in mice lacking the IKKalpha subunit of IkappaB kinase. Science.

[13] Senftleben U, Cao Y, Xiao G (2001). Activation by IKKalpha of a second, evolutionary conserved, NF-kappa B signaling pathway. Science.

[14] Dejardin E, Droin NM, Delhase M (2002). The lymphotoxin-beta receptor induces different patterns of gene expression via two NF-kappaB pathways. Immunity.

[15] Hayden MS, Ghosh S (2004). Signaling to NF-kappaB. Genes Dev.

[16] Bonizzi G, Karin M (2004). The two NF-kappaB activation pathways and their role in innate and adaptive immunity. Trends Immunol.

[17] Kinoshita D, Hirota F, Kaisho T (2006). Essential role of IkappaB kinase alpha in thymic organogenesis required for the establishment of self-tolerance. J Immunol.

[18] Takeda K, Takeuchi O, Tsujimura T (1999). Limb and skin abnormalities in mice lacking IKKalpha. Science.

[19] Sil AK, Maeda S, Sano Y, Roop DR, Karin M (2004). IkappaB kinase-alpha acts in the epidermis to control skeletal and craniofacial morphogenesis. Nature.

[20] Hu Y, Baud V, Oga T, Kim KI, Yoshida K, Karin M (2001). IKKalpha controls formation of the epidermis independently of NF-kappaB. Nature.

[21] Li Q, Lu Q, Bottero V (2005). Enhanced NF-{kappa}B activation and cellular function in macrophages lacking I{kappa}B kinase 1 (IKK1). Proc Natl Acad Sci USA.

[22] Lawrence T, Bebien M, Liu GY, Nizet V, Karin M (2005). IKKalpha limits macrophage NF-kappaB activation and contributes to the resolution of inflammation. Nature.

[23] Lu LF, Gondek DC, Scott ZA, Noelle RJ (2005). NF{kappa}B-inducing kinase deficiency results in the development of a subset of regulatory t cells, which shows a hyperproliferative activity upon glucocorticoid-induced TNF receptor family-related gene stimulation. J Immunol.

[24] Tas SW, Vervoordeldonk MJ, Hajji N (2007). Noncanonical NF-kappaB signaling in dendritic cells is required for indoleamine 2,3-dioxygenase (IDO) induction and immune regulation. Blood.

[25] Grohmann U, Volpi C, Fallarino F (2007). Reverse signaling through GITR ligand enables dexamethasone to activate IDO in allergy. Nat Med.

[26] Puccetti P, Grohmann U (2007). IDO and regulatory T cells: a role for reverse signalling and non-canonical NF-kappaB activation. Nat Rev Immunol.

[27] Grabiec AM, Tak PP, Reedquist KA (2008). Targeting histone deacetylase activity in rheumatoid arthritis and asthma as prototypes of inflammatory disease: should we keep our HATs on?. Arthritis Res Ther.

[28] Eberharter A, Becker PB (2002). Histone acetylation: a switch between repressive and permissive chromatin. Second in review series on chromatin dynamics. EMBO Rep.

[29] Ashburner BP, Westerheide SD, Baldwin AS Jr (2001). The p65 (RelA) subunit of NF-kappaB interacts with the histone deacetylase (HDAC) corepressors HDAC1 and HDAC2 to negatively regulate gene expression. Mol Cell Biol.

[30] Liu B, Yang Y, Chernishof V (2007). Proinflammatory stimuli induce IKKalpha-mediated phosphorylation of PIAS1 to restrict inflammation and immunity. Cell.

[31] Ghosh S, Hayden MS (2008). New regulators of NF-kappaB in inflammation. Nat Rev Immunol.

[32] Marok R, Winyard PG, Coumbe A (1996). Activation of the transcription factor nuclear factor-kappaB in human inflamed synovial tissue. Arthritis Rheum.

[33] Handel ML, McMorrow LB, Gravallese EM (1995). Nuclear factor-kappa B in rheumatoid synovium. Localization of p50 and p65. Arthritis Rheum.

[34] Foxwell B, Browne K, Bondeson J (1998). Efficient adenoviral infection with IkappaB alpha reveals that macrophage tumor necrosis factor alpha production in rheumatoid arthritis is NF-kappaB dependent. Proc Natl Acad Sci USA.

[35] Hiscott J, Marois J, Garoufalis J (1993). Characterization of a functional NF-kappa B site in the human interleukin 1 beta promoter: evidence for a positive autoregulatory loop. Mol Cell Biol.

[36] Fujisawa K, Aono H, Hasunuma T, Yamamoto K, Mita S, Nishioka K (1996). Activation of transcription factor NF-kappa B in human synovial cells in response to tumor necrosis factor alpha. Arthritis Rheum.

[37] Richmond A (2002). Nf-kappa B, chemokine gene transcription and tumour growth. Nat Rev Immunol.

[38] Chen CC, Rosenbloom CL, Anderson DC, Manning AM (1995). Selective inhibition of E-selectin, vascular cell adhesion molecule-1, and intercellular adhesion molecule-1 expression by inhibitors of I kappa B-alpha phosphorylation. J Immunol.

[39] Collins T, Read MA, Neish AS, Whitley MZ, Thanos D, Maniatis T (1995). Transcriptional regulation of endothelial cell adhesion molecules: NF-kappa B and cytokine-inducible enhancers. FASEB J.

[40] Mengshol JA, Vincenti MP, Coon CI, Barchowsky A, Brinckerhoff CE (2000). Interleukin-1 induction of collagenase 3 (matrix metalloproteinase 13) gene expression in chondrocytes requires p38, c-Jun N-terminal kinase, and nuclear factor kappaB: differential regulation of collagenase 1 and collagenase 3. Arthritis Rheum.

[41] Han Z, Boyle DL, Manning AM, Firestein GS (1998). AP-1 and NF-kappaB regulation in rheumatoid arthritis and murine collagen-induced arthritis. Autoimmunity.

[42] Wang CY, Mayo MW, Baldwin AS Jr (1996). TNF- and cancer therapy-induced apoptosis: potentiation by inhibition of NF-kappaB. Science.

[43] Van Antwerp DJ, Martin SJ, Kafri T, Green DR, Verma IM (1996). Suppression of TNF-alpha-induced apoptosis by NF-kappaB. Science.

[44] Miagkov AV, Kovalenko DV, Brown CE (1998). NF-kappaB activation provides the potential link between inflammation and hyperplasia in the arthritic joint. Proc Natl Acad Sci USA.

[45] Romashkova JA, Makarov SS (1999). NF-kappaB is a target of AKT in anti-apoptotic PDGF signalling. Nature.

[46] Wang CY, Mayo MW, Korneluk RG, Goeddel DV, Baldwin AS Jr (1998). NF-kappaB antiapoptosis: induction of TRAF1 and TRAF2 and c-IAP1 and c-IAP2 to suppress caspase-8 activation. Science.

[47] Yamamoto Y, Gaynor RB (2001). Therapeutic potential of inhibition of the NF-kappaB pathway in the treatment of inflammation and cancer. J Clin Invest.

[48] May MJ, D'Acquisto F, Madge LA, Glockner J, Pober JS, Ghosh S (2000). Selective inhibition of NF-kappaB activation by a peptide that blocks the interaction of NEMO with the IkappaB kinase complex. Science.

[49] Gerlag DM, Ransone L, Tak PP (2000). The effect of a T cell-specific NF-kappa B inhibitor on *in vitro* cytokine production and collagen-induced arthritis. J Immunol.

[50] Takada Y, Aggarwal BB (2004). Flavopiridol inhibits NF-kB activation induced by various carcinogens and inflammatory agents through inhibition of Ikappa Balpha kinase and p65 phosphorylation: Abrogation of cyclin D1, cyclooxygenase-2 and matrix metalloprotease-9. J Biol Chem.

[51] Eckmann L, Nebelsiek T, Fingerle AA (2008). Opposing functions of IKKbeta during acute and chronic intestinal inflammation. Proc Natl Acad Sci USA.

[52] Tak PP, Gerlag DM, Aupperle KR (2001). Inhibitor of nuclear factor kappaB kinase beta is a key regulator of synovial inflammation. Arthritis Rheum.

[53] Jimi E, Aoki K, Saito H (2004). Selective inhibition of NF-kappa B blocks osteoclastogenesis and prevents inflammatory bone destruction *in vivo*. Nat Med.

[54] Dai S, Hirayama T, Abbas S, Abu-Amer Y (2004). The IKK inhibitor, NEMO-binding domain peptide, blocks osteoclastogenesis and bone erosion in inflammatory arthritis. J Biol Chem.

[55] McIntyre KW, Shuster DJ, Gillooly KM (2003). A highly selective inhibitor of I kappa B kinase, BMS-345541, blocks both joint inflammation and destruction in collagen-induced arthritis in mice. Arthritis Rheum.

[56] Tas SW, Remans PH, Reedquist KA, Tak PP (2005). Signal transduction pathways and transcription factors as therapeutic targets in inflammatory disease: towards innovative antirheumatic therapy. Curr Pharm Des.

[57] Dasgupta S, Jana M, Zhou Y, Fung YK, Ghosh S, Pahan K (2004). Anti-neuroinflammatory effect of NF-kappaB essential modifier-binding domain peptides in the adoptive transfer model of experimental allergic encephalomyelitis. J Immunol.

[58] Murata T, Shimada M, Sakakibara S (2003). Discovery of novel and selective IKK-beta serine-threonine protein kinase inhibitors. Part 1. Bioorg Med Chem Lett.

[59] Castro AC, Dang LC, Soucy F (2003). Novel IKK inhibitors: beta-carbolines. Bioorg Med Chem Lett.

[60] Liang MC, Bardhan S, Li C, Pace EA, Porco JA Jr, Gilmore TD (2003). Jesterone dimer, a synthetic derivative of the fungal metabolite jesterone, blocks activation of transcription factor nuclear factor kappaB by inhibiting the inhibitor of kappaB kinase. Mol Pharmacol.

[61] Kobori M, Yang Z, Gong D (2004). Wedelolactone suppresses LPS-induced caspase-11 expression by directly inhibiting the IKK Complex. Cell Death Differ.

[62] Karin M, Yamamoto Y, Wang QM (2004). The IKK NF-kappa B system: a treasure trove for drug development. Nat Rev Drug Discov.

[63] Adriaansen J, Vervoordeldonk MJ, Tak PP (2006). Gene therapy as a therapeutic approach for the treatment of rheumatoid arthritis: innovative vectors and therapeutic genes. Rheumatology (Oxford).

[64] van de Loo FA, Smeets RL, van den Berg WB (2004). Gene therapy in animal models of rheumatoid arthritis: are we ready for the patients?. Arthritis Res Ther.

[65] Vervoordeldonk MJ, Aalbers C, Tak PP (2008). Advances in local targeted gene therapy for arthritis: towards clinical reality. Future Rheumatol.

[66] Vilaboa N, Voellmy R (2006). Regulatable gene expression systems for gene therapy. Curr Gene Ther.

[67] Goverdhana S, Puntel M, Xiong W (2005). Regulatable gene expression systems for gene therapy applications: progress and future challenges. Mol Ther.

[68] Saukkonen K, Hemminki A (2004). Tissue-specific promoters for cancer gene therapy. Expert Opin Biol Ther.

[69] Weber W, Fussenegger M (2006). Pharmacologic transgene control systems for gene therapy. J Gene Med.

[70] Bessis N, GarciaCozar FJ, Boissier MC (2004). Immune responses to gene therapy vectors: influence on vector function and effector mechanisms. Gene Ther.

[71] Raper SE, Chirmule N, Lee FS (2003). Fatal systemic inflammatory response syndrome in a ornithine transcarbamylase deficient patient following adenoviral gene transfer. Mol Genet Metab.

[72] Marshall E (1999). Gene therapy death prompts review of adenovirus vector. Science.

[73] Tas SW, Hajji N, Stenvers DJ, Firestein GS, Vervoordeldonk MJ, Tak PP (2006). Reduction of proinflammatory cytokine expression in the synovium by targeting IKKbeta *in vivo* in a rat model. Arthritis Rheum.

[74] Oitzinger W, Hofer-Warbinek R, Schmid JA, Koshelnick Y, Binder BR, de Martin R (2001). Adenovirus-mediated expression of a mutant IkappaB kinase 2 inhibits the response of endothelial cells to inflammatory stimuli. Blood.

[75] Catley MC, Sukkar MB, Chung KF (2006). Validation of the anti-inflammatory properties of small-molecule IkappaB Kinase (IKK)-2 inhibitors by comparison with adenoviral-mediated delivery of dominant-negative IKK1 and IKK2 in human airways smooth muscle. Mol Pharmacol.

[76] Chapoval SP, Al Garawi A, Lora JM (2007). Inhibition of NF-kappaB activation reduces the tissue effects of transgenic IL-13. J Immunol.

[77] Sanlioglu AD, Koksal IT, Karacay B, Baykara M, Luleci G, Sanlioglu S (2006). Adenovirus-mediated IKKbetaKA expression sensitizes prostate carcinoma cells to TRAIL-induced apoptosis. Cancer Gene Ther.

[78] Karacay B, Sanlioglu S, Griffith TS, Sandler A, Bonthius DJ (2004). Inhibition of the NF-kappaB pathway enhances TRAIL-mediated apoptosis in neuroblastoma cells. Cancer Gene Ther.

[79] Sanlioglu S, Luleci G, Thomas KW (2001). Simultaneous inhibition of Rac1 and IKK pathways sensitizes lung cancer cells to TNFalpha-mediated apoptosis. Cancer Gene Ther.

[80] Zhou LF, Yin KS, Zhu ZL (2005). Adenovirus-mediated overexpression of novel mutated IkappaBalpha inhibits nuclear factor kappaB activation in endothelial cells. Chin Med J (Engl ).

[81] Relic B, Bentires-Alj M, Ribbens C (2002). TNF-alpha protects human primary articular chondrocytes from nitric oxide-induced apoptosis via nuclear factor-kappaB. Lab Invest.

[82] Wheeler MD, Yamashina S, Froh M, Rusyn I, Thurman RG (2001). Adenoviral gene delivery can inactivate Kupffer cells: role of oxidants in NF-kappaB activation and cytokine production. J Leukoc Biol.

[83] Obara H, Takayanagi A, Hirahashi J (2000). Overexpression of truncated IkappaBalpha induces TNF-alpha-dependent apoptosis in human vascular smooth muscle cells. Arterioscler Thromb Vasc Biol.

[84] Griesenbach U, Scheid P, Hillery E (2000). Anti-inflammatory gene therapy directed at the airway epithelium. Gene Ther.

[85] Taylor BS, Shao L, Gambotto A, Ganster RW, Geller DA (1999). Inhibition of cytokine-induced nitric oxide synthase expression by gene transfer of adenoviral I kappa B alpha. Surgery.

[86] Hellerbrand C, Jobin C, Iimuro Y, Licato L, Sartor RB, Brenner DA (1998). Inhibition of NFkappaB in activated rat hepatic stellate cells by proteasome inhibitors and an IkappaB super-repressor. Hepatology.

[87] Jobin C, Panja A, Hellerbrand C (1998). Inhibition of proinflammatory molecule production by adenovirus-mediated expression of a nuclear factor kappaB super-repressor in human intestinal epithelial cells. J Immunol.

[88] Yang P, McKay BS, Allen JB, Roberts WL, Jaffe GJ (2003). Effect of mutant IkappaB on cytokine-induced activation of NF-kappaB in cultured human RPE cells. Invest Ophthalmol Vis Sci.

[89] Zhang HG, Huang N, Liu D (2000). Gene therapy that inhibits nuclear translocation of nuclear factor kappaB results in tumor necrosis factor alpha-induced apoptosis of human synovial fibroblasts. Arthritis Rheum.

[90] Bondeson J, Foxwell B, Brennan F, Feldmann M (1999). Defining therapeutic targets by using adenovirus: blocking NF-kappaB inhibits both inflammatory and destructive mechanisms in rheumatoid synovium but spares anti-inflammatory mediators. Proc Natl Acad Sci USA.

[91] Amos N, Lauder S, Evans A, Feldmann M, Bondeson J (2006). Adenoviral gene transfer into osteoarthritis synovial cells using the endogenous inhibitor IkappaBalpha reveals that most, but not all, inflammatory and destructive mediators are NFkappaB dependent. Rheumatology (Oxford).

[92] Bondeson J, Lauder S, Wainwright S (2007). Adenoviral gene transfer of the endogenous inhibitor IkappaBalpha into human osteoarthritis synovial fibroblasts demonstrates that several matrix metalloproteinases and aggrecanases are nuclear factor-kappaB-dependent. J Rheumatol.

[93] Ishiyama T, Dharmarajan S, Hayama M, Moriya H, Grapperhaus K, Patterson GA (2005). Inhibition of nuclear factor kappaB by IkappaB superrepressor gene transfer ameliorates ischemia-reperfusion injury after experimental lung transplantation. J Thorac Cardiovasc Surg.

[94] Trescher K, Bernecker O, Fellner B (2004). Adenovirus-mediated overexpression of inhibitor kappa B-alpha attenuates postinfarct remodeling in the rat heart. Eur J Cardiothorac Surg.

[95] Suetsugu H, Iimuro Y, Uehara T (2005). Nuclear factor {kappa}B inactivation in the rat liver ameliorates short term total warm ischaemia/reperfusion injury. Gut.

[96] Takahashi Y, Ganster RW, Ishikawa T (2001). Protective role of NF-kappaB in liver cold ischemia/reperfusion injury: effects of IkappaB gene therapy. Transplant Proc.

[97] Breuss JM, Cejna M, Bergmeister H (2002). Activation of nuclear factor-kappa B significantly contributes to lumen loss in a rabbit iliac artery balloon angioplasty model. Circulation.

[98] Bird MA, Black D, Lange PA, Samson CM, Hayden M, Behrns KE (2003). NFkappaB inhibition decreases hepatocyte proliferation but does not alter apoptosis in obstructive jaundice. J Surg Res.

[99] Schreiber J, Efron PA, Park JE, Moldawer LL, Barbul A (2005). Adenoviral gene transfer of an NF-kappaB super-repressor increases collagen deposition in rodent cutaneous wound healing. Surgery.

[100] Ni J, Takayama K, Ushijima R (2008). Adenovirus-mediated inhibitor kappaB gene transfer improves the chemosensitivity to anticancer drugs in human lung cancer *in vitro* and *in vivo*. Anticancer Res.

[101] Ni J, Takayama K, Inoshima N (2005). Gene transfer of inhibitor kappaB in human lung cancer cell line NCI-H460 inhibits tumorigenesis and angiogenesis *in vivo*. Anticancer Res.

[102] Li TJ, Jia LP, Gao XL, Huang AL (2006). Gene therapy that inhibits NF-kappaB results in apoptosis of human hepatocarcinoma by recombinant adenovirus. World J Gastroenterol.

[103] Tietze MK, Wuestefeld T, Paul Y (2000). IkappaBalpha gene therapy in tumor necrosis factor-alpha- and chemotherapy-mediated apoptosis of hepatocellular carcinomas. Cancer Gene Ther.

[104] Feig BW, Lu X, Hunt KK (1999). Inhibition of the transcription factor nuclear factor-kappa B by adenoviral-mediated expression of I kappa B alpha M results in tumor cell death. Surgery.

[105] Wang CY, Cusack JC Jr, Liu R, Baldwin AS Jr (1999). Control of inducible chemoresistance: enhanced anti-tumor therapy through increased apoptosis by inhibition of NF-kappaB. Nat Med.

[106] Bakker TR, Reed D, Renno T, Jongeneel CV (1999). Efficient adenoviral transfer of NF-kappaB inhibitor sensitizes melanoma to tumor necrosis factor-mediated apoptosis. Int J Cancer.

[107] Mukogawa T, Koyama F, Tachibana M (2003). Adenovirus-mediated gene transduction of truncated I kappa B alpha enhances radiosensitivity in human colon cancer cells. Cancer Sci.

[108] Weaver KD, Yeyeodu S, Cusack JC Jr, Baldwin AS Jr, Ewend MG (2003). Potentiation of chemotherapeutic agents following antagonism of nuclear factor kappa B in human gliomas. J Neurooncol.

[109] Chen S, Fribley A, Wang CY (2002). Potentiation of tumor necrosis factor-mediated apoptosis of oral squamous cell carcinoma cells by adenovirus-mediated gene transfer of NF-kappaB inhibitor. J Dent Res.

[110] Grimm D, Kay MA (2003). From virus evolution to vector revolution: use of naturally occurring serotypes of adeno-associated virus (AAV) as novel vectors for human gene therapy. Curr Gene Ther.

[111] Donsante A, Miller DG, Li Y (2007). AAV vector integration sites in mouse hepatocellular carcinoma. Science.

[112] Niemeyer GP, Herzog RW, Mount J (2009). Long term correction of inhibitor prone hemophilia B dogs treated with liver-directed AAV2 mediated factor IX gene therapy. Blood.

[113] Qu G, Bahr-Davidson J, Prado J (2007). Separation of adeno-associated virus type 2 empty particles from genome containing vectors by anion-exchange column chromatography. J Virol Methods.

[114] Ghivizzani SC, Gouze E, Gouze JN (2008). Perspectives on the use of gene therapy for chronic joint diseases. Curr Gene Ther.

[115] Kaiser J (2007). Clinical trials. Gene transfer an unlikely contributor to patient's death. Science.

[116] Adriaansen J, Tas SW, Klarenbeek PL (2005). Enhanced gene transfer to arthritic joints using adeno- associated virus type 5: implications for intra-articular gene therapy. Ann Rheum Dis.

[117] Apparailly F, Khoury M, Vervoordeldonk MJ (2005). Adeno-associated virus pseudotype 5 vector improves gene transfer in arthritic joints. Hum Gene Ther.

[118] Tas SW, Adriaansen J, Hajji N (2006). Amelioration of arthritis by intra-articular dominant negative IKKbeta gene therapy using adeno-associated virus type 5. Hum Gene Ther.

[119] Squadrito F, Deodato B, Squadrito G (2003). Gene transfer of IkappaBalpha limits infarct size in a mouse model of myocardial ischemia-reperfusion injury. Lab Invest.

[120] Squadrito F, Deodato B, Bova A (2003). Crucial role of nuclear factor-kappaB in neointimal hyperplasia of the mouse carotid artery after interruption of blood flow. Atherosclerosis.

[121] Makarov SS, Johnston WN, Olsen JC (1997). NF-kappa B as a target for anti-inflammatory gene therapy: suppression of inflammatory responses in monocytic and stromal cells by stable gene transfer of I kappa B alpha cDNA. Gene Ther.

[122] Meunier A, Latremoliere A, Dominguez E (2007). Lentiviral-mediated targeted NF-kappaB blockade in dorsal spinal cord glia attenuates sciatic nerve injury-induced neuropathic pain in the rat. Mol Ther.

[123] Morishita R, Sugimoto T, Aoki M (1997). *In vivo* transfection of cis element "decoy" against nuclear factor-kappaB binding site prevents myocardial infarction. Nat Med.

[124] Isomura I, Morita A (2006). Regulation of NF-kappaB signaling by decoy oligodeoxynucleotides. Microbiol Immunol.

[125] Morishita R, Tomita N, Kaneda Y, Ogihara T (2004). Molecular therapy to inhibit NFkappaB activation by transcription factor decoy oligonucleotides. Curr Opin Pharmacol.

[126] Ohtani K, Egashira K, Nakano K (2006). Stent-based local delivery of nuclear factor-kappaB decoy attenuates in-stent restenosis in hypercholesterolemic rabbits. Circulation.

[127] Egashira K, Suzuki J, Ito H, Aoki M, Isobe M, Morishita R (2008). Long-term follow up of initial clinical cases with NF-kappaB decoy oligodeoxynucleotide transfection at the site of coronary stenting. J Gene Med.

[128] Suzuki JI, Tezuka D, Morishita R, Isobe M (2009). An initial case of suppressed restenosis with nuclear factor-kappa B decoy transfection after percutaneous coronary intervention. J Gene Med.

[129] Tomita T, Takeuchi E, Tomita N (1999). Suppressed severity of collagen-induced arthritis by *in vivo* transfection of nuclear factor kappa B decoy oligodeoxynucleotides as a gene therapy. Arthritis Rheum.

[130] Kunugiza Y, Tomita T, Tomita N, Morishita R, Yoshikawa H (2006). Inhibitory effect of ribbon-type NF-kappaB decoy oligodeoxynucleotides on osteoclast induction and activity *in vitro* and *in vivo*. Arthritis Res Ther.

[131] Tomita T, Takano H, Tomita N (2000). Transcription factor decoy for NFkappaB inhibits cytokine and adhesion molecule expressions in synovial cells derived from rheumatoid arthritis. Rheumatology (Oxford).

[132] Shimizu H, Nakagami H, Tsukamoto I (2006). NFkappaB decoy oligodeoxynucleotides ameliorates osteoporosis through inhibition of activation and differentiation of osteoclasts. Gene Ther.

[133] Desmet C, Gosset P, Pajak B (2004). Selective blockade of NF-kappa B activity in airway immune cells inhibits the effector phase of experimental asthma. J Immunol.

[134] Fichtner-Feigl S, Fuss IJ, Preiss JC, Strober W, Kitani A (2005). Treatment of murine Th1- and Th2-mediated inflammatory bowel disease with NF-kappa B decoy oligonucleotides. J Clin Invest.

[135] De Vry CG, Prasad S, Komuves L (2007). Non-viral delivery of nuclear factor-kappaB decoy ameliorates murine inflammatory bowel disease and restores tissue homeostasis. Gut.

[136] Matsuda N, Hattori Y, Takahashi Y (2004). Therapeutic effect of *in vivo* transfection of transcription factor decoy to NF-kappaB on septic lung in mice. Am J Physiol Lung Cell Mol Physiol.

[137] Tomita N, Morishita R, Lan HY (2000). *In vivo* administration of a nuclear transcription factor-kappaB decoy suppresses experimental crescentic glomerulonephritis. J Am Soc Nephrol.

[138] Tomita N, Morishita R, Tomita S (2000). Transcription factor decoy for NFkappaB inhibits TNF-alpha-induced cytokine and adhesion molecule expression *in vivo*. Gene Ther.

[139] D'Acquisto F, Ialenti A, Ianaro A, Di Vaio R, Carnuccio R (2000). Local administration of transcription factor decoy oligonucleotides to nuclear factor-kappaB prevents carrageenin-induced inflammation in rat hind paw. Gene Ther.

[140] Nakamura H, Aoki M, Tamai K (2002). Prevention and regression of atopic dermatitis by ointment containing NF-kB decoy oligodeoxynucleotides in NC/Nga atopic mouse model. Gene Ther.

[141] Vos IH, Govers R, Grone HJ (2000). NFkappaB decoy oligodeoxynucleotides reduce monocyte infiltration in renal allografts. FASEB J.

[142] Azuma H, Tomita N, Kaneda Y (2003). Transfection of NFkappaB-decoy oligodeoxynucleotides using efficient ultrasound-mediated gene transfer into donor kidneys prolonged survival of rat renal allografts. Gene Ther.

[143] Ohmori K, Takeda S, Miyoshi S (2005). Attenuation of lung injury in allograft rejection using NF-kappaB decoy transfection-novel strategy for use in lung transplantation. Eur J Cardiothorac Surg.

[144] Kawamura I, Morishita R, Tomita N (1999). Intratumoral injection of oligonucleotides to the NF kappa B binding site inhibits cachexia in a mouse tumor model. Gene Ther.

[145] Kawamura I, Morishita R, Tsujimoto S (2001). Intravenous injection of oligodeoxynucleotides to the NF-kappaB binding site inhibits hepatic metastasis of M5076 reticulosarcoma in mice. Gene Ther.

[146] Azuma H, Tomita N, Sakamoto T (2008). Marked regression of liver metastasis by combined therapy of ultrasound-mediated NF kappaB-decoy transfer and transportal injection of paclitaxel, in mouse. Int J Cancer.

[147] Fire A, Xu S, Montgomery MK, Kostas SA, Driver SE, Mello CC (1998). Potent and specific genetic interference by double-stranded RNA in Caenorhabditis elegans. Nature.

[148] Fire AZ (2007). Gene silencing by double-stranded RNA (Nobel Lecture). Angew Chem Int Ed Engl.

[149] Mello CC (2007). Return to the RNAi world: rethinking gene expression and evolution (Nobel Lecture). Angew Chem Int Ed Engl.

[150] Elbashir SM, Harborth J, Lendeckel W, Yalcin A, Weber K, Tuschl T (2001). Duplexes of 21-nucleotide RNAs mediate RNA interference in cultured mammalian cells. Nature.

[151] Gewirtz AM (2007). On future's doorstep: RNA interference and the pharmacopeia of tomorrow. J Clin Invest.

[152] Corey DR (2007). Chemical modification: the key to clinical application of RNA interference?. J Clin Invest.

[153] Akhtar S, Benter IF (2007). Nonviral delivery of synthetic siRNAs *in vivo*. J Clin Invest.

[154] Grimm D, Kay MA (2007). Therapeutic application of RNAi: is mRNA targeting finally ready for prime time?. J Clin Invest.

[155] de Fougerolles AR (2008). Delivery vehicles for small interfering RNA *in vivo*. Hum Gene Ther.

[156] Lee UJ, Choung SR, Prakash KV (2008). Dual knockdown of p65 and p50 subunits of NF-kappaB by siRNA inhibits the induction of inflammatory cytokines and significantly enhance apoptosis in human primary synoviocytes treated with tumor necrosis factor-alpha. Mol Biol Rep.

[157] Lianxu C, Hongti J, Changlong Y (2006). NF-kappaBp65-specific siRNA inhibits expression of genes of COX-2, NOS-2 and MMP-9 in rat IL-1beta-induced and TNF-alpha-induced chondrocytes. Osteoarthritis Cartilage.

[158] Tian F, Zang WD, Hou WH, Liu HT, Xue LX (2006). Nuclear factor-kB signaling pathway constitutively activated in esophageal squamous cell carcinoma cell lines and inhibition of growth of cells by small interfering RNA. Acta Biochim Biophys Sin (Shanghai).

[159] Duan J, Friedman J, Nottingham L, Chen Z, Ara G, Van Waes C (2007). Nuclear factor-kappaB p65 small interfering RNA or proteasome inhibitor bortezomib sensitizes head and neck squamous cell carcinomas to classic histone deacetylase inhibitors and novel histone deacetylase inhibitor PXD101. Mol Cancer Ther.

[160] Lun M, Zhang PL, Pellitteri PK, Law A, Kennedy TL, Brown RE (2005). Nuclear factor-kappaB pathway as a therapeutic target in head and neck squamous cell carcinoma: pharmaceutical and molecular validation in human cell lines using Velcade and siRNA/NF-kappaB. Ann Clin Lab Sci.

[161] Guo J, Verma UN, Gaynor RB, Frenkel EP, Becerra CR (2004). Enhanced chemosensitivity to irinotecan by RNA interference-mediated down-regulation of the nuclear factor-kappaB p65 subunit. Clin Cancer Res.

[162] Braun T, Carvalho G, Coquelle A (2006). NF-kappaB constitutes a potential therapeutic target in high-risk myelodysplastic syndrome. Blood.

[163] Inoue M, Matsumoto S, Saito H, Tsujitani S, Ikeguchi M (2008). Intraperitoneal administration of a small interfering RNA targeting nuclear factor-kappa B with paclitaxel successfully prolongs the survival of xenograft model mice with peritoneal metastasis of gastric cancer. Int J Cancer.

[164] Austin RL, Rune A, Bouzakri K, Zierath JR, Krook A (2008). siRNA-mediated reduction of inhibitor of nuclear factor-kappaB kinase prevents tumor necrosis factor-alpha-induced insulin resistance in human skeletal muscle. Diabetes.

[165] Chen LX, Lin L, Wang HJ (2008). Suppression of early experimental osteoarthritis by *in vivo* delivery of the adenoviral vector-mediated NF-kappaBp65-specific siRNA. Osteoarthritis Cartilage.

[166] Pinkenburg O, Platz J, Beisswenger C, Vogelmeier C, Bals R (2004). Inhibition of NF-kappaB mediated inflammation by siRNA expressed by recombinant adeno-associated virus. J Virol Methods.

[167] Mi J, Zhang X, Rabbani ZN (2008). RNA aptamer-targeted inhibition of NF-kappa B suppresses non-small cell lung cancer resistance to doxorubicin. Mol Ther.

[168] Gupta S, Young D, Maitra RK (2008). Prevention of cardiac hypertrophy and heart failure by silencing of NF-kappaB. J Mol Biol.

[169] van Duivenvoorde LM, Louis-Plence P, Apparailly F (2004). Antigen-specific immunomodulation of collagen-induced arthritis with tumor necrosis factor-stimulated dendritic cells. Arthritis Rheum.

[170] Tarner IH, Slavin AJ, McBride J (2003). Treatment of autoimmune disease by adoptive cellular gene therapy. Ann N Y Acad Sci.

[171] Morita Y, Yang J, Gupta R (2001). Dendritic cells genetically engineered to express IL-4 inhibit murine collagen-induced arthritis. J Clin Invest.

[172] Figdor CG, de Vries IJ, Lesterhuis WJ, Melief CJ (2004). Dendritic cell immunotherapy: mapping the way. Nat Med.

[173] Haniffa MA, Wang XN, Holtick U (2007). Adult human fibroblasts are potent immunoregulatory cells and functionally equivalent to mesenchymal stem cells. J Immunol.

[174] Yang J, Bernier SM, Ichim TE (2003). LF15-0195 generates tolerogenic dendritic cells by suppression of NF-kappaB signaling through inhibition of IKK activity. J Leukoc Biol.

[175] Tan PH, Sagoo P, Chan C (2005). Inhibition of NF-kappaB and oxidative pathways in human dendritic cells by antioxidative vitamins generates regulatory T cells. J Immunol.

[176] Tomasoni S, Aiello S, Cassis L (2005). Dendritic cells genetically engineered with adenoviral vector encoding dnIKK2 induce the formation of potent CD4+ T-regulatory cells. Transplantation.

[177] Aiello S, Cassis P, Cassis L (2007). DnIKK2-transfected dendritic cells induce a novel population of inducible nitric oxide synthase-expressing CD4+. Transplantation.

[178] Li M, Zhang X, Zheng X (2007). Immune modulation and tolerance induction by RelB-silenced dendritic cells through RNA interference. J Immunol.

[179] Bonham CA, Peng L, Liang X (2002). Marked prolongation of cardiac allograft survival by dendritic cells genetically engineered with NF-kappa B oligodeoxyribonucleotide decoys and adenoviral vectors encoding CTLA4-Ig. J Immunol.

[180] Adema GJ, de Vries IJ, Punt CJ, Figdor CG (2005). Migration of dendritic cell based cancer vaccines: *in vivo* veritas?. Curr Opin Immunol.

